# 조기진통 사정 알고리즘은 실습 시 조기진통 관련 지식, 임상수행자신감, 교육만족도에 유효한가?: 유사실험 연구

**DOI:** 10.4069/kjwhn.2023.08.17

**Published:** 2023-09-26

**Authors:** Hee-Young Choi, Jeung-Im Kim

**Affiliations:** 1 School of Nursing, Graduate School of Soonchunhyang University, Cheonan, Korea; 1 순천향대학교 일반대학원 간호학과; 2 School of Nursing, Soonchunhyang University College of Medicine, Cheonan, Korea; 2 순천향대학교 의과대학 간호학과

**Keywords:** Algorithms, Clinical competence, Knowledge, Premature obstetric labor, 알고리즘, 임상수행, 지식, 조기진통

## Abstract

**Purpose:**

Preterm birth is increasing, and obstetric nurses should have the competency to provide timely care. Therefore, training is necessary in the maternal nursing practicum. This study aimed to investigate the effects of practice education using a preterm-labor assessment algorithm on preterm labor-related knowledge and clinical practice confidence in senior nursing students.

**Methods:**

A pre-post quasi-experimental design with three groups was used for 61 students. The preterm-labor assessment algorithm was modified into three modules from the preterm-labor assessment algorithm by March of Dimes. We evaluated preterm labor-related knowledge, clinical practice confidence, and educational satisfaction. Data were analyzed with the paired t-test and repeated-measures analysis of variance.

**Results:**

The practice education using a preterm-labor assessment algorithm significantly improved both preterm labor-related knowledge and clinical practice confidence (paired t=–7.17, *p*<.001; paired t=–5.51, *p*<.001, respectively). The effects of the practice education using a preterm-labor assessment algorithm on knowledge lasted until 8 weeks but decreased significantly at 11 and 13 weeks after the program, while the clinical practice confidence significantly decreased at 8 weeks post-program.

**Conclusion:**

The practice education using a preterm-labor assessment algorithm was effective in improving preterm labor-related knowledge and clinical practice confidence. The findings suggest that follow-up education should be conducted at 8 weeks, or as soon as possible thereafter, to maintain knowledge and clinical confidence, and the effects should be evaluated.

## Introduction

임상간호 실습교육은 임상현장에서 일하게 될 간호학생의 대처능력 및 비판적 사고능력과 임상수행능력을 향상시키며[1], 이론 지식을 실무에 적용할 수 있도록 훈련하는 중요한 교과과정이다. 그러나 임상실습지인 병원은 환자의 안전 및 권리를 우선하므로, 간호학생이 환자에게 직접 간호를 수행하는 것을 제한하는 경우가 많이 있다[2]. 이에 따라 간호대학에서는 임상에서 직접 환자에게 적용하기 어려운 술기나 집중적인 훈련이 필요한 사례들을 중심으로 시뮬레이션 실습교육을 하고 있다[3].

분만실 실습이 어려운 상황에서[4] 고위험 임산부 관리를 위한 집중치료실(maternal-fetal intensive care unit, MFICU)이 마련된 점은 바람직한 방향이지만, 임산부의 중증도가 높아 간호학생은 대상자를 만나는 것조차 어렵다. 고위험 임신 합병증 중 조기진통은 임신 34–36주는 중등도, 임신 34주 미만은 중증으로 분류되며 37주 미만의 조산은 고위험 분만에 해당되는데, 모자보건 의료 종사자들은 고위험 임산부를 잘 찾아내는 것이 중요하다[5].

조기진통 임부를 구별해내기 위해서는, 임신 37주 이전이면서 자궁경부의 개대와 소실을 동반하고 규칙적인 자궁 수축, 생리통 같은 통증, 질 분비물의 증가, 골반 압박감 등의 증상[6]이 있는지 평가할 필요가 있다. 또한, 자궁 수축보다 자궁경부의 변화가 먼저 일어나거나[7], 자궁경부 변화 없이 자궁 수축만 계속되는 조기진통도 있을 수 있으므로[8], 분만실 간호사는 정상 임부의 증상과는 다른 조기진통 임부가 보이는 증상을 구별하기 위한 건강 사정을 해야 한다. 그리고 산과 의사에게 즉각적인 보고가 필요한 조산위험 상황인지 혹은 추가적인 검사가 이루어져야 할 상황인지에 따라 체계적으로 수행하여야 한다[9,10].

분만실 간호사가 체계적인 수행을 하기 위해서는 자궁경관 개대 정도와 자궁경관 길이 및 조기 양막 파수 여부에 따른 임상적 결정을 할 수 있는 훈련[11]이 필요하며, 이는 간호학생 때부터 실습교육에서 다룰 필요가 있다. 분만실 간호사들이 조산위험 여부에 대한 임상적 의사결정을 내리는 데 도움이 되는 가이드라인으로 조기진통 사정 도구(Preterm Labor Assessment Toolkit, PLAT)가 있다. 이는 의료의 질 향상과 임상적 의사결정을 위해 질병과 증상 관리 과정을 시각화한 임상 가이드라인으로[11], 신속한 의사결정이 이루어지도록 지원할 수 있어[12] 임상경험이 적은 간호사에게 유용하다[13]. 그러므로 아직 임상경험이 적고 고위험인 조기진통 임부를 간호한 경험이 거의 없는 간호학생들에게도 도움이 될 것으로 예상되나, 임상에서 직접 적용하기는 어려우므로 시뮬레이션 실습에서 PLAT를 적용하여 조기진통 관리를 숙련할 필요가 있다.

한편 간호학생을 위한 분만 관련 시뮬레이션 교육은 임상적 의사결정을 돕는 구체적인 지침보다는 정상분만 과정 및 산욕기 간호 모듈로서 팀 기반 학습을 적용한 시뮬레이션 교육[14], 정상분만 간호에 대한 이론 교육 후 3주 뒤 시나리오와 표준화 환자를 활용한 시뮬레이션 교육[15]들이다. 이론 교육에서 분만 과정을 배우지만, 자궁경부 개대 3 cm 이상 여부, 자궁경관 길이 20 mm 미만 여부, 태아 섬유결합소(fetal fibronectin, fFN) 양성 여부와 같은 요소를 포함하여 임상적 의사결정을 하는 알고리즘 교육은 찾아 보기 어렵다.

MFICU 간호사 역량강화 교육 프로그램을 운영해온 Kim [16]은 PLAT에 제시된 조기진통 사정 알고리즘을 국내에 발표한 후, MFICU 간호사를 위한 교육자료로 사용해 왔다[17]. 조기진통 위험 사정을 통해 적시에 필요한 중재를 하기 위해서는 ‘자궁경부 개대 3 cm 이상’, ‘fFN 양성’, ’20 mm 미만의 짧은 자궁경관 길이’의 기준[11]을 명확히 알고 이 알고리즘에 따른 관리를 하는 훈련이 필요하다. 특히 MFICU 실습의 기회를 갖지 못하는 간호학생들에게는 조기진통을 사정하는 알고리즘 교육이 유익할 것으로 생각된다. 그러므로 먼저 알고리즘을 적용한 단기간의 실습교육이 조기진통 관련 지식과 임상수행자신감 향상에 기여하는지 살펴볼 필요가 있다. 또한, 단기교육의 지속 효과는 4주 혹은 8주로 보고되고 있어[18,19] 본 연구에서도 알고리즘을 적용한 실습교육의 지속 효과를 함께 평가할 필요가 있다.

이에 본 연구에서는 조기진통의 발생기전 및 임상증상과 조기진통 사정 알고리즘에 대한 교육을 제공하고 이를 통합 수준의 시뮬레이션 실습교육에 적용함으로써(이하 조기진통 알고리즘 적용 실습이라고 함) 간호학생의 조기진통 관련 지식과 임상수행자신감에 미치는 효과를 평가하고자 하며, 구체적인 연구목적은 다음과 같다.

첫째, 조기진통 알고리즘 적용이 조기진통 관련 지식과 임상수행자신감에 미치는 단기 효과를 비교한다.

둘째, 조기진통 알고리즘 적용이 조기진통 관련 지식과 임상수행자신감에 미치는 지속 효과를 비교한다.

셋째, 조기진통 알고리즘 적용 실습교육에 대한 교육 만족도를 평가한다.

## Methods

**Ethical statement:** This study was conducted as part of clinical practicum and was exempted from the Institutional Review Board of Soonchunhyang University (No. 202106-SB-062-02). All procedures adhered to the principles of the Declaration of Helsinki.

### 연구 설계

본 연구는 조기진통 알고리즘 적용 실습의 단기 효과와 지속 효과를 파악하기 위한 사전-사후 유사실험 연구(pre-post quasi-experimental design)이다. 본 연구는 Table 1과 같이 정해진 실습 스케줄에 따른 통상적인 교육과정에 의해 설정된 3개 그룹에 조기진통 알고리즘 중재 처치를 제공하고 사전조사와 사후조사를 각 3회씩 실시하였다. 교육의 지속 효과 평가 시기는 기존 시뮬레이션 교육 효과 연구에서 8주에 실시한 설계[18,19]를 참고하여, 본 연구에서는 그룹 3이 교육 후 8주가 되는 시점에서 2차 사후조사를 실시하였다.

### 연구 절차

실험군인 그룹 1, 2, 3은 2021년 9월 1일부터 10월 7일까지 7주(추석연휴 주간 미운영) 사이에 각 그룹당 2일, 그룹은 2주 간격을 두고 순차적으로 통합실습 실습 일정에 따라 진행하였다. 실습 전 시점(T0)에 사전조사(Y1, Y2), 실습 후 시점(T1)에 사후조사(Y3, Y4, Y7)가 이루어졌다. 알고리즘 적용 실습교육 효과의 지속성 평가를 위한 2차 사후조사(Y5, Y6)는 그룹 3이 실습을 종료한 후 8주차가 되는 시점(T2)인 2021년 12월 2일에 실시하였다. 자료 수집 방법은 온라인 Google 설문 양식을 통해 진행하였으며 소요시간은 약 20분 정도이다(Table 1).

### 연구 대상

본 연구의 대상은 간호학과 4학년 1학기에 여성건강간호학 이론 수업과 임상실습(분만실 혹은 산부인과 병동)을 마친 학생으로서, 연구 당시 4학년 2학기 재학 중이며, 동일 학기에 ‘통합실습 2’ 교과목에서 1가지 모듈을 2주 간격으로 실시하는 4가지 모듈 중 하나로서, 32주 5일 임부여성의 임상증상 표현 ‘배가 조여요’ 모듈을 수행하는 학생이다. 통상적인 교육의 일환으로 진행하기 때문에 4학년 학생 전원(63명)을 대상으로 하였다. 평가 당일 COVID-19 확진으로 2명이 제외되어 최종 연구 대상자는 61명이 되었다. 이는 G-power 3.1.9.7 for Windows 프로그램을 활용, 효과 크기(d)=0.4, 유의수준(α)=0.05, 검정력(1–β)=.80으로 산출된 표본 수는 52명과 탈락률 10%를 고려한 표본 수 58명을 충족했다. 실습 조 편성 및 일정은 실습교육 코디네이터에 의해 작성되었으며, 4가지 모듈 실습 중 분만 모듈은 3개조로 편성하였고 조별 대상자 수는 Table 1과 같다.

### 연구 도구

#### 조기진통 관련 지식

본 연구에서는 여성건강간호학 교재[6]를 바탕으로 조기진통 산모 간호 시 필수적인 핵심지식을 평가하기 위해 정상분만과 차별화되는 조기진통의 사정 및 간호에 관한 지식을 설문 문항으로 작성하였다. 이는 2021년 고위험 MFICU 간호사 보수교육에서 참석자들의 지식 변화를 알아보기 위해 개발된 20문항에 대해, 조기진통이나 조산과 관련된 문항으로 적합한지 여성건강간호학 교수 7인에게 도구의 내용 타당도를 검토받았다. 6–10인의 전문가일 경우 내용타당도 지수(content validity index) .78 이상이 권장되어[20], 본 연구에서는 .80 이상인 15문항을 선정하였다. 각 문항에 대하여 정답은 1점, 오답은 0점으로 처리하였고 점수(가능 범위, 0–15)가 높을수록 지식 점수가 높은 것을 의미하며, 본 연구에서 KR-20 신뢰도는 .78로 나타났다.

#### 임상수행자신감

본 연구에서 임상수행자신감은 간호사를 대상으로 개발된 자가보고형 임상수행능력 평가도구인 6영역 척도(six-dimension scale) [21]를 간호대학생에게 적용한 도구[22]의 문항 중 본 연구에 맞지 않았던 문항을 수정하였고, 개발자와 번안자에게 모두 사용 승인을 받았다. 수정한 이유는 기존 문항에서 간호 대상 및 간호수행과 관련된 내용이 명확하게 기술되어 있지 않고 모호하였기 때문이다. 본 연구에서 수정한 사항은 간호 대상에 환자, 가족을 포함하고 간호수행 내용에 활력징후의 정확한 항목들, 투약 시 5 right, 간호 문제를 추가하였다. 도구의 문항 수는 변경없이 원래의 도구에서 정한 바와 같이 간호과정 5문항, 간호 술기 5문항, 교육 및 협력 5문항 총 15문항이며, ‘매우 못한다’ 1점에서 ‘매우 잘한다’ 5점으로, 점수(가능 범위, 15–75)가 높을수록 임상수행자신감이 높음을 의미한다. 기존 연구의 Cronbach’s α는 .94 [22]이었고, 본 연구에서도 Cronbach’s α가 .94로 나타났다.

#### 교육 만족도

본 연구에서 조기진통 알고리즘 시뮬레이션 실습교육에 대한 만족도 조사는 Song과 Hong [23]이 개발한 역할극을 통한 임상교육 만족도를 수정한 10문항으로 측정하였다. 수정된 부분은 문항의 주어를 ‘의사-환자 역할극’에서‘조기진통 증상 분류를 이용한 시뮬레이션 수업’으로 변경하였고, 간호대학생을 대상으로 조사하기 위해 ‘진단능력 향상’, ‘비내시경 기술 향상’, ‘면담능력 향상’등의 문항을 제외한 총 10문항으로 구성하였다. 5점 Likert 척도로 ‘전혀 그렇지 않다’ 1점, ‘매우 그렇다’ 5점으로 측정하여 점수(가능 범위, 10–50)가 높을수록 조기진통 알고리즘 시뮬레이션 실습교육 만족도가 높음을 의미한다. 도구 개발 당시 신뢰도는 Cronbach’s α .88, 본 연구에서는 Cronbach’s α .96이었다.

### 연구 중재: 조기진통 사정 알고리즘 교육과 적용

#### 조기진통 사정 알고리즘 교육자료 내용 확정

조기진통 사정 알고리즘 교육자료는 PLAT에 포함된 조기진통 분류 알고리즘[11]을 본 연구자가 한국어로 번역•수정한 후 여성건강간호학 교과목을 20년 이상 담당해온 교수의 감수를 받았다. 양막 파수를 확인할 수 있는 임상적 진단 방법 중 의료기관별로 시행 여부가 달라지는 검사인 Ferning, Amnisure와 그 외 배양검사인 group B streptococcus culture, bacterial vaginosis screen 등은 제외하였다. 간호대학생의 수준에서 학습 및 적용 가능하도록 ‘임부와 태아 상태를 조산사 또는 담당의에게 알린다’는 내용은 ‘담당의에게 알린다’로 수정하였다.

#### 조기진통 알고리즘 적용을 위한 교육과 훈련

전체적인 교육 흐름 및 시간은 Supplementary Table 1과 같이 이루어졌다.

##### (1) 교육 내용과 교육 방법

각 그룹은 평가일 전날 오후 3시부터 ‘조기진통 알고리즘 적용을 위한 교육’을 받았다. 교육 내용은 PLAT의 내용을 이해할 수 있도록 조기진통의 정의, 조산 위험성 사정의 필요성, fFN 양성의 의미와 해석, 자궁경부 길이를 측정하는 의미와 해석, 무자극검사(non-stress test) 결과 해석에 따른 조기진통 사정 알고리즘, 알고리즘에 따른 조산 위험성에 대한 임상적 판단, 조산 위험성 분류에 따른 투약, 산소 투여 등의 중재로 구성되었다.

이 때 조산 위험성은 PLAT에 제시된 기준을 따라 임부가 호소하는 임상증상, 양막 파수 여부, 자궁경부 개대 정도, fFN 검사 결과, 자궁경관 길이를 통해 임상적 판단을 하게 되는데, 다음과 같이 고위험(high risk), 불분명(equivocal), 저위험(low risk)으로 나뉜다. 이를 바탕으로 예시 자료를 이용하여 32주 5일인 임신 여성의 세 가지 임상 상황별로 판단할 사항과 판단하는 근거를 교육하였다. COVID-19 범유행 상황이어서 평가일 전날 시청각 자료(Power Point)를 활용하여 온라인(Zoom)을 통해 교육을 제공하였다(Supplementary Table 1).

• 상황 ① 자궁경부 3 cm 개대

→평가: 다른 조건과 관계없이 경부 개대가 3 cm 이상이면 고위험으로 판정

• 상황 ② 자궁경부 1 cm 개대, 자궁경관 길이 19 mm

→ 평가: 경관 길이가 20 mm 미만이므로 경부 개대가 3 cm 미만일지라도 고위험으로 판정

• 상황 ③ 자궁경부 1 cm 개대, 자궁경관 길이 22 mm, fFN (+)

→ 평가: 경부 개대가 2 cm 미만이고 자궁경관 길이가 20 mm 이상이므로 불분명으로 판단하여 경과 관찰을 함. fFN (+) 결과는 1주일 이내 분만 예측의 참조치에 해당

(※경부 개대 2 cm 이상이고 fFN (+)이면 다른 조건과 관계없이 고위험으로 분류)

##### (2) 알고리즘 기반 시나리오 작성

실습 학생들은 교육받은 조기진통에 대한 이론 교육과 세 가지 상황별 알고리즘을 반영하여 시나리오와 간호계획을 작성하였다. 시나리오는 사전에 공지한 조별로 소그룹 방에서 90분간 토의하며 작성하도록 하였다. 시나리오 작성 후 시나리오를 제출하도록 하였고 연구자와 공동 연구자 2명이 시나리오의 적절성 여부를 검토하여 피드백을 제공하였다.

##### (3) 알고리즘 적용 연습

실습 학생들은 알고리즘 기반 시나리오를 바탕으로 상황별 판단을 적용하는 훈련을 하였다. 알고리즘 적용에 필요한 술기로 질 내진과 양수파막검사(pH paper [UNIV pH 1–11] for nitrazine test)를 환자 시뮬레이터인 SimMom 3G (Laerdal, Stavanger, Norway)를 대상으로 연습하였다. 두 가지 검사 모두 알고리즘을 통해 검사항목과 결과를 연결해 보기 위한 목적으로 실시하였으며, 학생들이 작성한 조기진통 관리 알고리즘에 관한 조별 역할극을 간호사 1과 간호사 2의 2인 1조로, 1개 조 당 30분씩 진행하였다.

##### (4) 알고리즘 적용 실습(Supplementary Figure 1–4)

알고리즘 적용은 SimMom 3G, 표준화 환자, 지속적인 모니터링이 이뤄지는 분만 시뮬레이션 환경에서 이루어졌다. 양수파막검사는 간호학생의 “아래로 흐르는 느낌이 있나요?”라는 질문에 표준화 환자는 “그런 느낌이 없어요”로 답하도록 하였다. SimMom 3G는 상황별 임부의 심박수, 호흡 횟수, SpO_2_, 태아 심박수, 자궁 수축 곡선과 자궁 수축 시 자궁 내 압력 등의 변화 값을 지속적으로 모니터링하는 기기로 사용하였고, 표준화 환자는 사전에 평가 대본을 숙지하도록 하였으며 평가 당일 아침에 훈련 대본을 보면서 특정 시점에서 증상을 호소하거나 간호학생에게 질문하도록 하였다. 세 가지 상황을 반영한 3개의 코팅 카드를 가지고 실습 학생이 내진 행위를 수행하면 3가지 상황 중 하나의 상황에 맞는 자궁경부 개대 정도와 경관 길이를 알려주었다. 이 때 세 가지 상황에 따라 그 범위 안에 있는 기준으로 연구자와 공동 연구자 2명이 정보를 제공하면 학생들은 그 정보에 맞는 알고리즘의 임상경로를 따라 필요한 행위를 하도록 훈련하였다.

간호학생들은 모니터에 나타난 자궁 수축 시 자궁 내 압력과 표준화 환자의 진통 사정을 통한 자궁 수축 특성을 통합하여 담당교수에게 보고하면, 교수는 세 가지 임상 상황별 지시사항을 알리고 필요한 처치를 하도록 하였다. 고위험일 경우 자궁 수축 억제제 투여를 지시하였고, 이 약물의 부작용으로 고혈당을 의심하여 혈당검사를 하면 교수가 혈당 수치를 불러주었으며, SPO_2_가 90% 미만으로 떨어지는 경우에는 산소 투여 등의 과정이 이루어지도록 진행하였다. 고위험을 판단하기에 자궁 수축 특성이 불분명할 경우 계속 관찰한 후 다시 알고리즘 적용 상황이 발생하는지 판단하고 사정을 반복하도록 하였다. 알고리즘 적용 실습이 끝난 학생은 녹화된 본인의 영상을 확인하도록 하였다.

##### (5) 디브리핑(Debriefing)

전체 알고리즘 적용 실습이 끝난 후 해당 그룹의 실습학생, 표준화 환자, 연구자, 공동 연구자가 함께 알고리즘 적용 실습에서 느낀 점에 대해 의견을 나누었으며 수행 과정을 분석하고 성찰하는 시간을 가졌다. 디브리핑에 활용한 성찰일지 문항은 ‘알고리즘 적용에서 어려운 점은 무엇이었나요?’와 ’시뮬레이션 실습이 끝난 후 알고리즘이 어떻게 도움이 되었나요?’의 총 2문항으로 학생들이 자유롭게 기술하도록 하였다. 표준화 환자는 서울-경기 Clinical Performance Examination 컨소시엄에서 활동 중이며 3년 동안 참여해주고 있는 출산 경험이 있는 여성으로, 2주 전에 상황, 시나리오 흐름도가 포함된 표준화 환자 훈련 대본을 제공하였고 실습 당일 추가적으로 회의를 하여 반응과 표현을 숙지하고 있었다. 표준화 환자는 디브리핑에도 참여하여 개선방향을 설명하여 주었다.

### 자료 분석 방법

본 연구에서 수집된 자료는 IBM SPSS ver. 26.0 (IBM Corp., Armonk, NY, USA)을 이용하여 분석하였다.

1) 연구 대상자의 일반적 특성과 교육 만족도는 빈도, 백분율, 평균 및 표준편차로 파악하였다.

2) 조기진통 알고리즘 적용 실습 전후 조기진통 관련 지식과 임상수행자신감의 변화, 실습 종료 후 교육 효과는 대응표본 t검정(paired t-test), 그룹 간 비교는 분산분석(analysis of variance, ANOVA), 알고리즘 적용 실습교육의 지속 효과는 반복 측정(repeated-measures) ANOVA로 분석하였다.

## Results

### 대상자의 일반적 특성

본 연구에 참여한 대상자의 평균 연령은 23.6 (±2.53)세로 25세 이하 47명(77.0%), 26세 이상 14명(23.0%)이었다. 대상자 중 여학생은 58명(95.1%), 남학생은 3명(4.9%)이었다. ‘간호과정과 비판적사고’ 교과목 성적은 평균 85.0 (±7.45)점, 4학년 1학기 여성건강간호학 1 성적은 평균 87.0 (±7.35)점이었다. 1학기 동안 분만실 실습은 33명(54.1%), 산부인과 실습은 28명(45.9%)이었다.

### 조기진통 알고리즘 적용 실습의 단기 효과

#### 조기진통 관련 지식

조기진통 알고리즘 적용 실습교육이 조기진통 관련 지식에 미친 단기 효과를 파악하기 위해 T0에서 세 그룹의 동질성을 평가한 결과, 유의한 차이가 없어(F=0.86, *p*=.430) T0 시점에서 세 그룹의 동질성이 확인되었다. 전체 대상자의 교육 전과 후의 조기진통 관련 지식의 평균점수는 T0 시점(9.97±1.80)보다 T1 시점(12.16±1.74)에서 통계적으로 유의하게 증가하였다(t=–7.17, *p*<.001). 또한, 각 그룹별 실습교육 전후 차이를 살펴본 결과, 그룹 1 (t=–4.77, *p*<.001), 그룹 2 (t=–4.10, *p*<.001), 그룹 3 (t=–3.55, *p*=.002) 모두 지식 점수가 유의하게 상승하였다(Table 2).

#### 임상수행자신감

조기진통 알고리즘 적용 실습교육이 임상수행자신감에 미친 효과를 파악하기 위해 T0에서 세 그룹의 동질성을 평가한 결과, 유의한 차이가 없어 세 그룹의 동질성이 확인되었다(F=0.83, *p*=.441). 전체 대상자의 교육 전후 임상수행자신감의 평균점수는 T0 시점(52.70±6.87)보다 T1 시점(59.77±7.59)에서 통계적으로 유의하게 증가하였다(t=–5.51, *p*<.001). 또한, 각 그룹별 실습교육 전후 차이를 살펴본 결과, 그룹 1 (t=–3.42, *p*=.003), 그룹 2 (t=–2.32, *p*=.032), 그룹 3 (t=–3.88, *p*=.001) 모두 임상수행자신감이 유의하게 상승하였다(Table 3).

### 조기진통 알고리즘 적용 실습교육의 지속 효과

**Figure 1.**
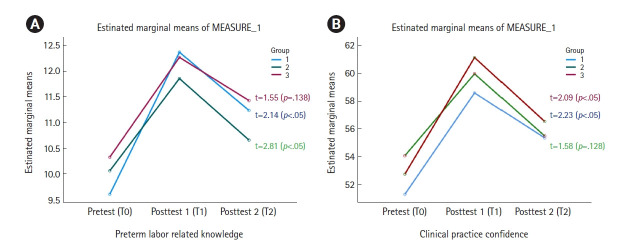
Changes in three groups over time (t-value: T1–T2 difference). (A) Preterm labor-related knowledge. (B) Clinical practice confidence.

조기진통 알고리즘 적용 실습의 지속 효과는 마지막 그룹 3의 실습교육이 끝나고 8주차가 되는 시점(그룹 2: 11주째, 그룹 1: 13주째)에서 전체 실습학생을 대상으로 이루어졌으며 그 결과는 Figure 1과 같다.

#### 조기진통 관련 지식

조기진통 관련 지식에 있어 시간 경과에 따른 알고리즘 실습교육의 지속 효과를 반복 측정 ANOVA로 살펴본 결과, 각 시점에서 세 그룹 간 유의한 차이가 없었으나 세 그룹 각각의 평균점수는 유의하게 증가하였다(F=11.64, *p*=.001). Bonferroni 사후검정 결과 조기진통 관련 지식 점수는 T0 시점보다 T1 시점에서 유의하게 높았고, T0 시점보다 T2 시점의 지식 점수가 유의하게 높았다. 그러나, T1보다는 T2 시점에서 지식이 유의하게 감소하였다(Table 4). 그룹별로 T1, T2 시점의 조기진통 관련 지식의 평균점수를 비교한 결과, 그룹 3은 실습교육 8주째인 T2 시점의 평균점수와 T1 시점의 평균점수 사이에 유의한 차이가 없었다(t=1.55, *p*=.138). 그룹 2는 교육 후 11주째인 T2 시점의 평균점수가 T1 시점보다 유의하게 감소하였고(t=2.81, *p*<.05), 그룹 1은 교육 후 13주째인 T2 시점의 평균점수가 T1 시점보다 유의하게 감소하였다(t=2.14, *p*<.05) (Figure 1A).

#### 임상수행자신감

임상수행자신감에 있어 시간 경과에 따른 알고리즘 실습교육의 지속 효과를 반복 측정 ANOVA로 살펴본 결과, 각 시점에서 세 그룹간 유의한 차이가 없었으나 세 그룹의 각각의 평균점수는 유의하게 증가하였다(F=19.45, *p*<.001). Bonferroni 사후검정 결과 임상수행자신감은 T0 시점보다 T1 시점에 유의하게 높았고, T0 시점보다 T2 시점 점수가 유의하게 높게 나타났다. 그러나 T2시점의 임상수행자신감은 T1 시점보다 유의하게 감소하였다(Table 4). 그룹별로 T1, T2 시점의 임상수행자신감의 평균점수를 비교한 결과, 그룹 3은 교육 후 8주째인 T2 시점의 평균점수가 T1 시점보다 유의하게 감소하였다(t=2.09, *p*<.05). 그룹 2는 교육 후 11주째인 T2 시점의 평균점수가 T1 시점보다 유의하게 감소하였고(t=2.23, *p*<.05), 그룹 1은 교육 후 13주째인 T2 시점의 평균점수가 T1 시점과 비교하여 감소 경향을 보였으나 *p*<.05수준에서는 유의하지 않았다(t=1.58, *p*=.128) (Figure 1B).

### 조기진통 알고리즘 적용 실습교육 만족도

실습학생의 모두 4학년 2학기 재학생으로, 조기진통 알고리즘 적용 시뮬레이션 실습교육 만족도는 실습 직후에 평균 45.30 (±5.35)점으로 전체적인 만족도가 높았다. 세부 문항별로 살펴보았을 때 ‘앞으로 후배들의 실습교육으로 조기진통 증상 분류를 이용한 시뮬레이션 수업을 하는 것에 찬성한다.’가 최고점이었고(4.62±0.61), ‘조기진통 증상 분류를 이용한 시뮬레이션 수업을 통하여 조기진통 산모 간호에 자신감을 얻었다.’가 최하점(4.21)으로 모든 문항에서 4점 이상으로 나타났다(Supplementary Table 2).

## Discussion

본 연구는 PLAT의 조기진통 사정 알고리즘을 적용한 후 조기진통 관련 지식과 임상수행자신감에 미치는 단기 효과와 교육 프로그램의 지속 효과 및 교육 만족도를 평가하였다. 이에 본 연구 결과를 바탕으로 조기진통 알고리즘 적용 실습의 효과에 대하여 논하고자 한다.

먼저 본 연구 결과 조기진통 알고리즘 적용 실습은 실습 전과 비교하였을 때 실습 직후 조기진통 관련 지식, 임상수행자신감을 유의하게 향상시켰다. 이는 프로토콜을 기반으로 한 알고리즘이 지식을 향상시킨다는 연구[24], 알고리즘 기반 교육 프로그램이 간호사의 지식 향상에 효과가 있다는 연구[25]와 시뮬레이션 실습교육 후 지식이 향상되었다는 연구[26]와 맥을 같이 하는 결과라고 평가할 수 있다. 또한 본 연구에서 교육 후 임상수행자신감이 향상된 결과는, 알고리즘 기반의 상부 위장관 출혈 간호 시나리오를 간호대학생을 대상으로 시뮬레이션 교육 후 간호수행자신감, 만족도가 향상되었다는 연구 결과[27]와 유사하였다. 간호 제공자마다 일관성이 있으며, 정확하고 시기 적절한 간호중재를 제공할 수 있는 알고리즘이 임상 판단력을 향상시키고[28], 임상 판단력이 높을수록 간호수행자신감이 향상된다는[29] 연구들을 지지하는 결과이다. 본 연구에서도 임신 37주의 의미가 조산을 결정하는 기준이라는 점, 임신 37주가 되지 않은 임부에서 자궁경관 3 cm 개대는 3 cm 미만과 달리 다른 어떤 조건과 관계없이 조산으로 진행될 고위험 분만 상황이라는 점, fFN 검사 결과 양성은 조기진통이 일어날 가능성이 높고 조산으로 이어질 수 있는 결과라는 알고리즘을 미리 익히고, 유사한 상황에서 그 알고리즘을 적용하는 실습을 통해 임상 판단력과 임상수행자신감을 향상하는 데 기여하였을 것으로 생각한다.

다음으로 조기진통 알고리즘 적용 실습교육의 조기진통 관련 지식의 지속 효과를 분석한 결과, 그룹 3 (알고리즘 적용 실습교육 후 8주째)에서만 교육 직후 점수와 비교하여 유의한 감소가 없는 것으로 나타나 교육 후 8주까지 교육 효과가 지속된 것으로 추정할 수 있다. 반면, 그룹 2 (알고리즘 적용 실습교육 후 11주째)와 그룹 1 (알고리즘 적용 실습교육 후 13주째)은 조기진통 관련 지식이 교육 직후와 비교하였을 때 통계적으로 유의하게 감소하였다. 그러나 세 그룹 모두 실습교육 전 지식 점수와 비교하여 유의한 감소가 없었다. 즉 알고리즘 실습교육을 하는 것이 하지 않았을 때보다 짧게는 8주, 길게는 13주까지 지식에 주는 교육 효과가 지속된다고 보여진다. 알고리즘 교육의 지속 효과에 관한 선행연구가 거의 없어 간호학생의 심폐소생술 교육의 지속성을 살펴보았다. 심폐소생술 교육 후 8주까지는 지식이 유지되었지만 6개월 후에 유의하게 감소하는 결과[30]에 비추어 볼 때, 교육 후 8–13주에 재교육이 필요할 것으로 보인다. 이는 간호사의 산후출혈 시뮬레이션 교육 효과가 9개월 후에 사전 결과와 비슷한 수준으로 감소하였다는 결과[31]와 비교할 때, 교육 대상자가 학생인가 간호사인가에 따라 다를 수 있다는 점, 기억해야 할 내용의 양, 해당 교육 외 결과에 영향을 줄 수 있는 요인들을 더 탐색하여 재검토할 필요가 있다.

한편, 임상수행자신감의 경우 본 연구에서는 그룹 3 (알고리즘 적용 실습교육 후 8주째)과 그룹 2 (알고리즘 적용 실습교육 후 11주째)에서 통계적으로 유의한 감소가 있었으나, 그룹 1 (알고리즘 적용 실습교육 후 13주째)은 감소 경향은 보였으나 유의하지는 않았다. 그러나 세 그룹 모두 실습교육 전보다는 유의한 감소가 없었다. 즉 알고리즘 실습교육을 하는 것이 하지 않았을 때보다 짧게는 8주, 길게는 13주까지 임상수행자신감에 미치는 교육 효과가 지속되는 것으로 보인다. 본 연구 결과는 수행자신감이 8주까지는 유지되었으나 6개월 후에 유의하게 감소한다는 결과[30]와 비교할 때 교육 후 13주–6개월 사이에 어느 시점에서 재교육이 필요할지에 대한 후속 연구가 필요하다. 아울러 임상수행자신감 향상에 기여하기 위해서는 표준화된 가이드라인과 프로토콜[25], 일관성이 있고 정확하며 시기 적절한 간호중재를 제공할 수 있는 알고리즘[28]이어야 한다는 점에서 추후 연구에서는 국내 전문가들과 함께 표준화된 알고리즘 프로토콜을 업데이트할 필요가 있다.

다음으로 본 연구에서 조기진통 알고리즘 시뮬레이션 실습교육 만족도는 평균 45.30점으로 비교적 높은 점수를 보였다. 이는 조산사의 주산기 시뮬레이션 교육의 만족도 평가에서 시뮬레이션 실습 후 임상추론 및 임상적 의사결정과 관련된 항목이 가장 낮았던 결과[32]와 비교할 때 임상적 의사결정을 돕는 알고리즘이 실습교육 만족도를 높일 수 있었다고 추정된다. 또한, 본 연구에서 만족도 세부 항목 중 ‘앞으로 후배들의 실습교육으로 조기진통 증상 분류를 이용한 시뮬레이션 수업을 하는 것에 찬성한다’가 4.62점으로 가장 높은 점수를 나타내어 조기진통 사정 알고리즘을 적용한 시뮬레이션 실습교육을 계속할 필요가 있을 것으로 생각한다.

본 연구에서는 간호대학생에게 처음으로 PLAT의 조기진통 사정 알고리즘을 적용하고 이 알고리즘 실습교육의 단기 효과와 지속 효과를 기술하였으나 다음과 같은 제한점이 있다. 첫째, 본 연구는 통상적인 교육과정 속에 이루어졌기 때문에 실습교육 적용 시기와 조사 시기 등을 무작위 실험설계로 진행하지 못한 간헐적 시계열 설계로서, 조기진통 사정 알고리즘의 교육 효과를 비교할 대조군이 없다는 제한점이 있다. 둘째, 본 연구에서는 3개조로 나누어 기간이 달랐던 점과 지속 효과를 평가하는 시기가 교육 후 8주차, 11주차, 13주차와 같이 일치하지 않았다. 이에 본 연구 결과는 시점별 해석에 주의를 기울일 필요가 있으며, 추후 연구에서는 이러한 제한점을 고려한 연구설계와 반복 연구를 제안한다. 또한 본 연구에서는 조기진통 임부의 임신 주수를 지정하였으나 조기진통 임부의 임신 주수의 변화를 부여하고 자궁경부 봉축술과 같은 조건을 포함한 알고리즘을 개발할 것을 제안한다. 한편 본 연구에서 효과크기 d=0.4를 채택하였으나 d=0.25 [30], d=0.5 [2], d=0.6 [25]과 같이 다양하여 연구설계와 효과크기 측면의 검토가 필요하다. 

이러한 제한점에도 불구하고 본 연구는 자궁경부 개대 및 자궁경관 길이, fFN 검사 결과를 포함한 PLAT의 조기진통 사정 알고리즘을 적용하고 이에 대한 단기 효과와 지속 효과를 확인한 국내 최초의 연구로서 앞으로 더 개발할 필요성을 보여주었다는 의의가 있다고 생각한다. 또한, 본 연구의 대상자는 4학년 2학기 재학생으로 정규 교과과정에서는 조기진통의 이론 수업을 듣지 않았던 학생이었음에도 불구하고 알고리즘 적용 실습교육이 효과적이었므로, 실습교육 기간을 좀 더 늘리고 적절한 시기에 재교육을 실시한다면 조기진통 관련 지식과 임상수행자신감이 더 오래 지속될 것으로 생각된다.

결론적으로 본 연구 결과를 통해 조기진통 사정 알고리즘을 적용한 실습교육이 간호학생의 지식과 임상수행자신감을 향상시키는 효과적인 교육 방법이 될 수 있음을 밝혔다는 의의가 있다. 알고리즘을 통해 지식을 시나리오 상황에 적용하는 임상 판단 과정을 훈련해 봄으로써 간호학생의 임상 역량을 향상시켜 장래에 분만실 신규 간호사가 되었을 때 임상수행자신감을 갖고 안전한 출산에 기여할 수 있기를 기대한다.

## Figures and Tables

**Table 1.** Study design and process

Experimental group	Pretest (T0)	Intervention	Posttest (T1)	2nd posttest (T2)
Group 1 (n=22)	Y1, Y2	X	Y3, Y4, Y7	Y5, Y6 (13th week after posttest)
Group 2 (n=20)	Y1, Y2	X	Y3, Y4, Y7	Y5, Y6 (11th week after posttest)
Group 3 (n=19)	Y1, Y2	X	Y3, Y4, Y7	Y5, Y6 (8th week after posttest)

Y1, Y3, Y5: preterm labor-related knowledge; Y2, Y4, Y6: clinical practice confidence; Y7: educational satisfaction.

**Table 2.** Changes in preterm labor-related knowledge over time (N=61)

Group	Mean±SD		Paired t	*p*
	Pretest (T0)	Posttest 1 (T1)		
Total	9.97±1.80	12.16±1.74	–7.17	<.001
Group 1 (n=22)	9.59±1.68	12.36±2.17	–4.77	<.001
Group 2 (n=20)	10.05±1.61	11.85±1.42	–4.10	<.001
Group 3 (n=19)	10.32±2.11	12.26±1.52	–3.55	.002
F (*p*)	0.86 (.430)	0.49 (.615)		

**Table 3.** Changes in clinical practice confidence over time (N=61)

Group	Mean±SD		Paired t	*p*
	Pretest (T0)	Posttest 1 (T1)		
Total	52.70±6.87	59.77±7.59	–5.51	<.001
Group 1 (n=22)	51.36±6.89	58.55±8.05	–3.42	.003
Group 2 (n=20)	54.10±7.57	59.90±7.47	–2.32	.032
Group 3 (n=19)	52.79±6.07	61.05±7.36	–3.88	.001
F (*p*)	0.83 (.441)	0.55 (.579)		

**Table 4.** Repeated-measures ANOVA for preterm labor related knowledge and clinical practice confidence (N=61)

Variable	Time	Mean±SD	Sum of squares	F (*p*)	Bonferroni
Knowledge	T0	9.99 ± 0.23^a^	37.70	11.64 (.001)	
	T1	12.16 ± 0.23^b^			a<b, a<c, b>c
	T2	11.10 ± 0.25^c^			
Confidence	T0	52.70 ± 6.87^d^	281.87	19.45 (<.001)	d<e, d<f, e>f
	T1	59.77 ± 7.59^e^			
	T2	55.77 ± 7.00^f^			

ANOVA: analysis of variance; CI: confidence interval.

## Supplementary materials

Further details on supplementary materials are presented online (available at https://doi.org/10.4069/kjwhn.2023.08.17).

Supplementary Table 1. Training program based on preterm labor assessment algorithm

Supplementary Table 2. Satisfaction on simulation training using preterm labor-assessment algorithm (N=61)

Supplementary Figure 1. Practice education based on preterm labor assessment algorithm.

Supplementary Figure 2. Preterm labor assessment algorithm: Preterm labor is identified.

Supplementary Figure 3. Preterm labor assessment algorithm: Equivocal results.

Supplementary Figure 4. Preterm labor assessment algorithm: Low risk of preterm labor.
