# Supplementary material for: Does a preterm labor-assessment algorithm improve preterm labor-related knowledge, clinical practice confidence, and educational satisfaction?: a quasi-experimental study
Source: Korean J Women Health Nurs. 2023 Sep 26;29(3):219–28. [Article in Korean] doi: 10.4069/kjwhn.2023.08.17 (PMC10565533; doi:10.4069/kjwhn.2023.08.17)
Supplement: Supplementary Table 1. — Training program based on preterm labor assessment algorithm [file kjwhn-2023-08-17-Supplementary-Table-1.pdf]

Supplementary Table 1. Training program based on preterm labor assessment algorithm

Day	Contents	Methods/Place	Time (min)
1st day	**Algorithm-based education for preterm labor assessment (PLA)**	Lecture/online	20
	• Definition and symptoms of preterm-labor (PTL) and the importance of assessing Preterm Birth (PTB) risk		
	• Basic concepts of PLA		
	• Diagnostic tests within PLA		
	• Methods of assessing PTB risk using PLA		
	• Nursing interventions for PTL in pregnant women based on the degree of PTB risk		
	• Medication therapy used for PTL Women		
	**Orientation and cases**	Introduction/online	10
	• Explanation of the practicum schedule and procedure for PLA women		
	• Grouping of teams (2 students/team); Introduction of PTL cases		
	**Algorithm-based scenario development**	Team discussion/online	90
	• Development of PTL nursing scenarios based on the PLA algorithm and role assignment		
2nd day	**Role play**		
	• Role-playing exercises and practice based on scenarios created by the teams	Practice/women health nursing practice room	30
	**Evaluation**	Simulation room	25
	**Debriefing**	Debriefing room	60
