# Supplementary material for: Does a preterm labor-assessment algorithm improve preterm labor-related knowledge, clinical practice confidence, and educational satisfaction?: a quasi-experimental study
Source: Korean J Women Health Nurs. 2023 Sep 26;29(3):219–28. [Article in Korean] doi: 10.4069/kjwhn.2023.08.17 (PMC10565533; doi:10.4069/kjwhn.2023.08.17)
Supplement: Supplementary Table 2. — Satisfaction on simulation training using preterm labor-assessment algorithm (N=61) [file kjwhn-2023-08-17-Supplementary-Table-2.pdf]

Supplementary Table 2. Satisfaction on simulation training using preterm labor-assessment algorithm (N=61)

Questions	Mean ± SD
1) 조기진통 증상분류를 이용한 시뮬레이션 수업은 흥미로웠다.	4.54 ± 0.65
2) 나는 조기진통 증상분류를 이용한 시뮬레이션 수업에 적극적으로 참여하였다.	4.59 ± 0.62
3) 조기진통 증상분류를 이용한 시뮬레이션 수업 이후 조기진통 간호에 대한 관심이 증가하였다.	4.51 ± 0.60
4) 조기진통 증상분류를 이용한 시뮬레이션 수업은 적절하다고 생각한다.	4.57 ± 0.59
5) 앞으로 후배들의 실습교육으로 조기진통 증상분류를 이용한 시뮬레이션 수업을 하는 것에 찬성한다.	4.62 ± 0.61
6) 조기진통 증상분류를 이용한 시뮬레이션 수업이 나의 임상 지식에 도움이 되었다,	4.61 ± 0.58
7) 조기진통 증상분류를 이용한 시뮬레이션 수업을 통하여 나의 임상 지식 상태를 파악하는 데 유익하였다.	4.57 ± 0.62
8) 조기진통 증상분류를 이용한 시뮬레이션 수업을 통하여 조기진통 산모 간호에 자신감을 얻었다,	4.21 ± 0.82
9) 조기진통 증상분류를 이용한 시뮬레이션 수업은 나의 실제 상황에 대한 대응 능력에 도움이 될 것이다,	4.52 ± 0.62
10) 조기진통 증상분류를 이용한 시뮬레이션 수업은 미래 간호사로서 환자를 치료할 때 도움이 될 것이다.	4.54 ± 0.56
Total	45.30 ± 5.35

SD, standard deviation.
