# Supplementary material for: Does a preterm labor-assessment algorithm improve preterm labor-related knowledge, clinical practice confidence, and educational satisfaction?: a quasi-experimental study
Source: Korean J Women Health Nurs. 2023 Sep 26;29(3):219–28. [Article in Korean] doi: 10.4069/kjwhn.2023.08.17 (PMC10565533; doi:10.4069/kjwhn.2023.08.17)
Supplement: Supplementary Figure 1. — Practice education based on preterm labor assessment algorithm. [file kjwhn-2023-08-17-Supplementary-Fig-1.pdf]

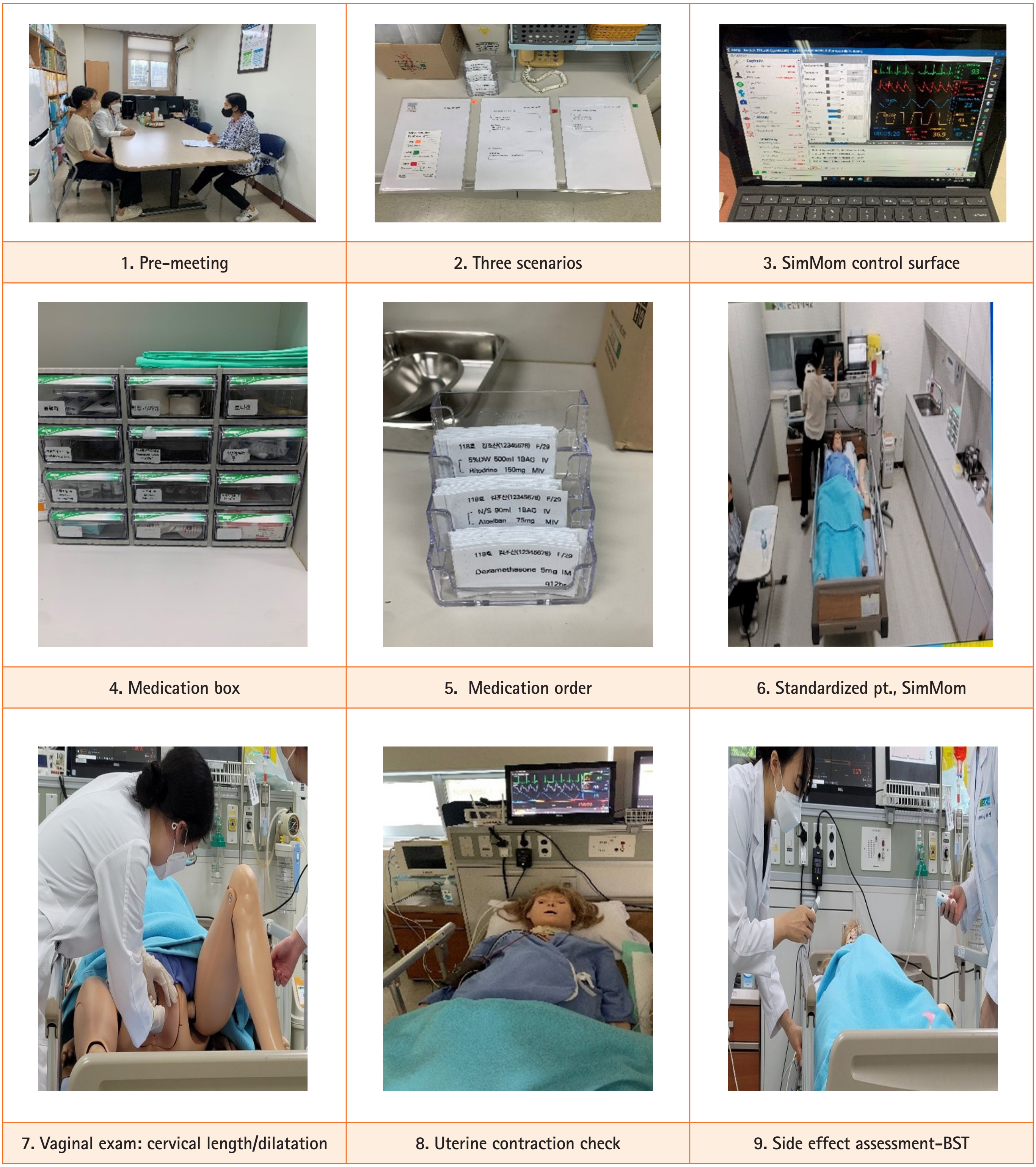

Supplementary Figure 1. Practice education based on preterm labor assessment algorithm.
