# Supplementary figures and images for: Does a preterm labor-assessment algorithm improve preterm labor-related knowledge, clinical practice confidence, and educational satisfaction?: a quasi-experimental study
Source: Korean J Women Health Nurs. 2023 Sep 26;29(3):219–28. [Article in Korean] doi: 10.4069/kjwhn.2023.08.17 (PMC10565533; doi:10.4069/kjwhn.2023.08.17)

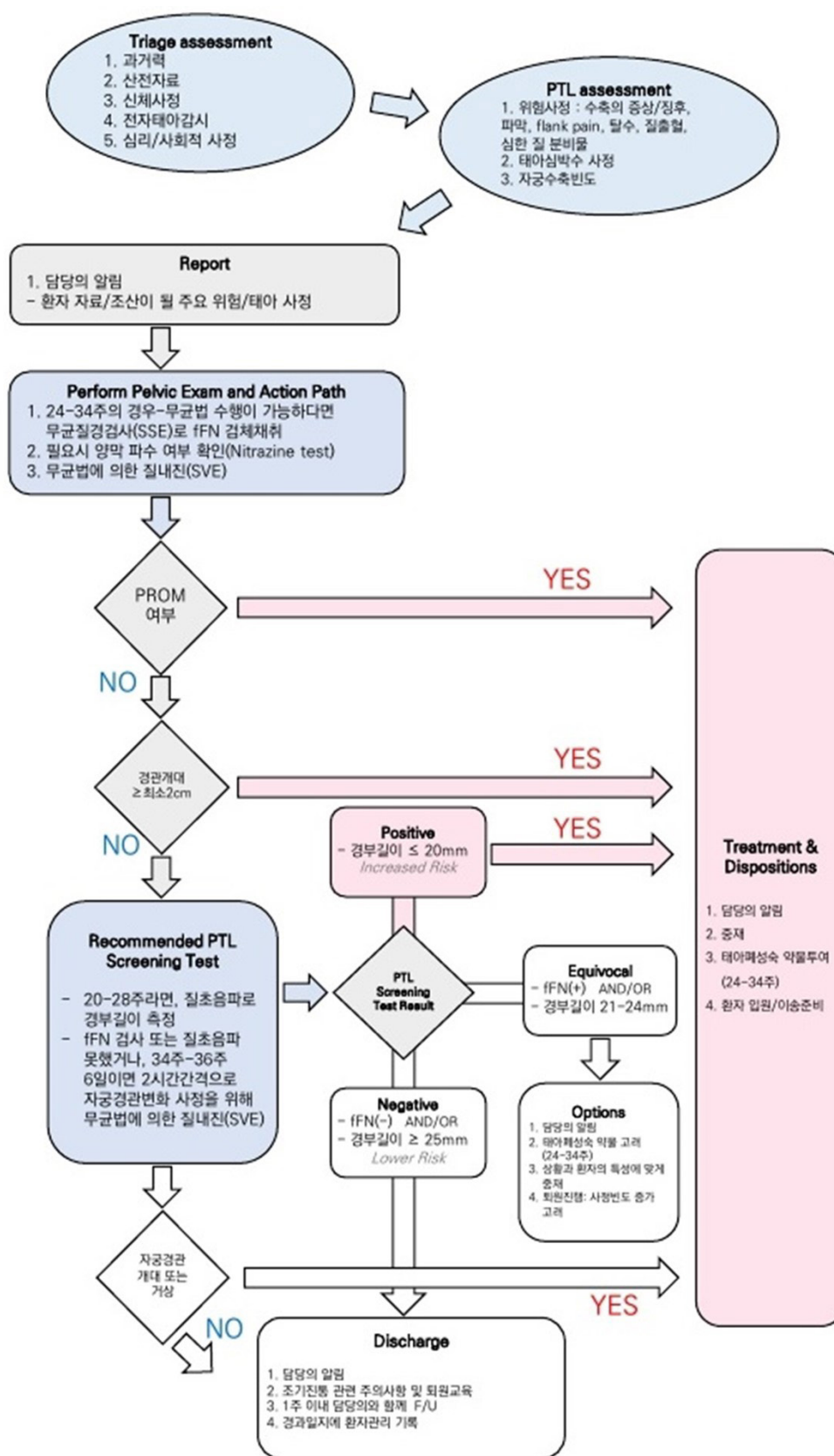

Supplementary Figure 2. Preterm labor assessment algorithm: Preterm labor is identified.

Supplement: Supplementary Figure 2. — Preterm labor assessment algorithm: Preterm labor is identified. [file kjwhn-2023-08-17-Supplementary-Fig-2.pdf]

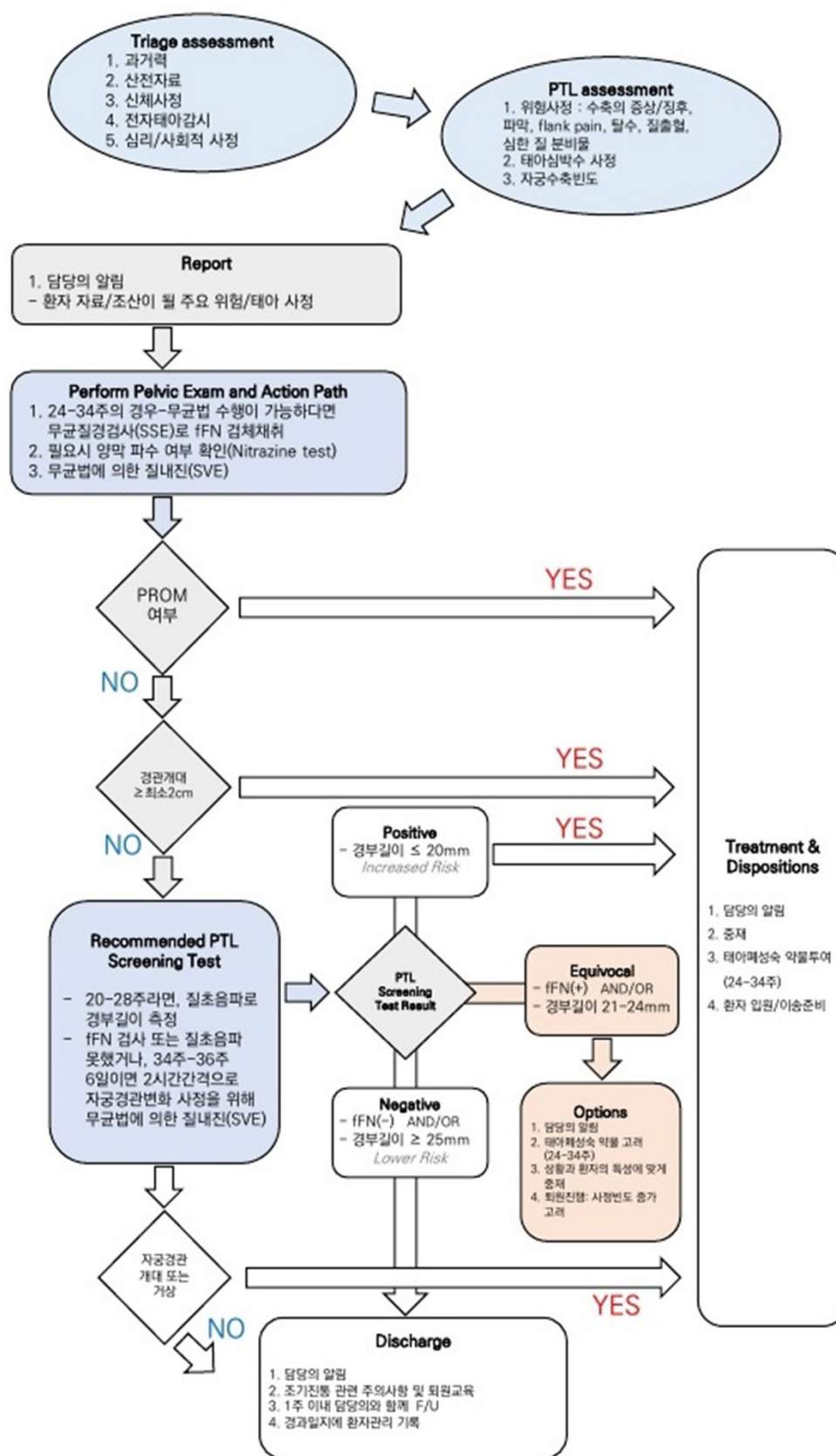

Supplementary Figure 3. Preterm labor assessment algorithm: Equivocal results.

Supplement: Supplementary Figure 3. — Preterm labor assessment algorithm: Equivocal results. [file kjwhn-2023-08-17-Supplementary-Fig-3.pdf]

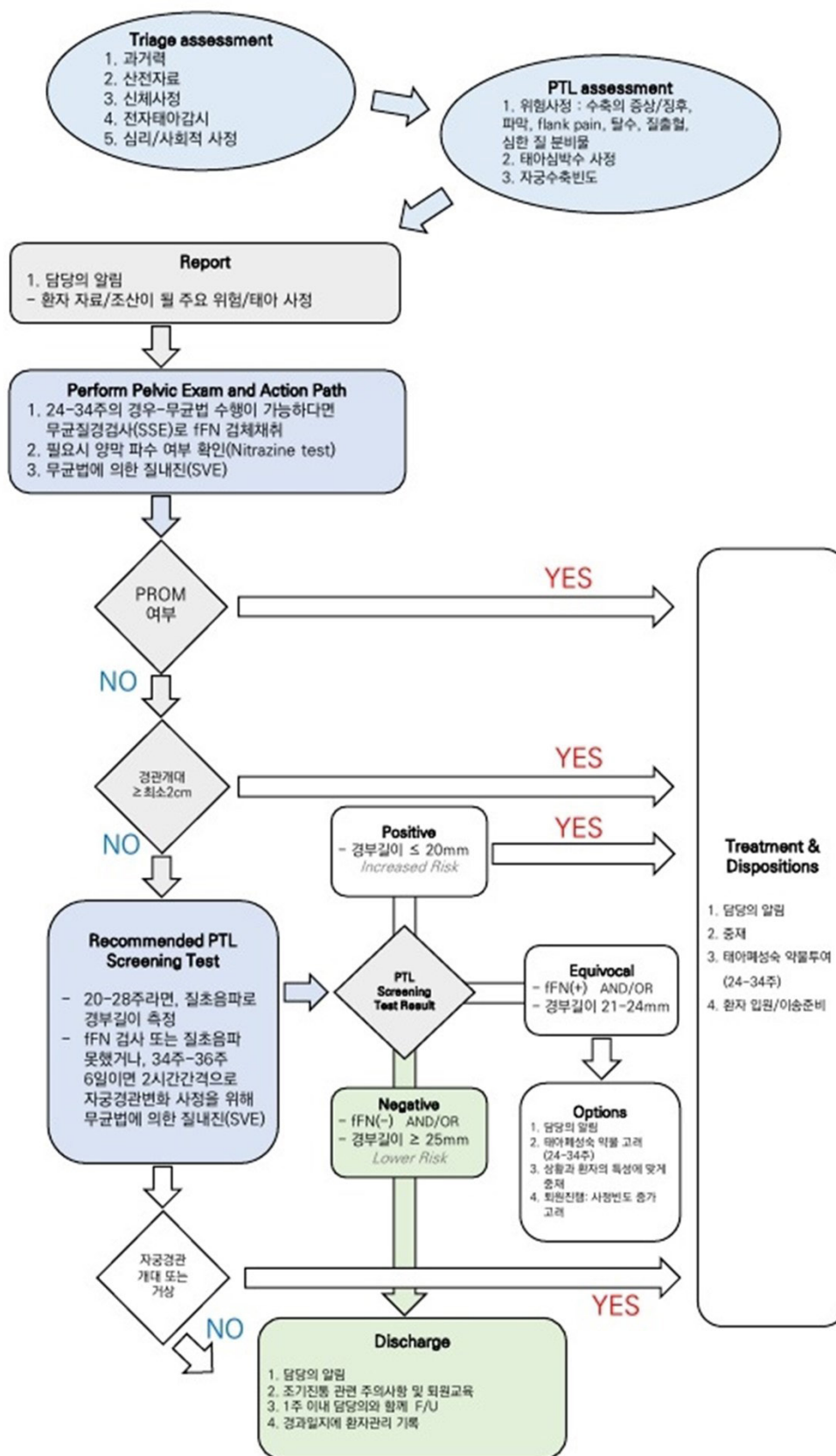

Supplementary Figure 4. Preterm labor assessment algorithm: Low risk of preterm labor.

Supplement: Supplementary Figure 4. — Preterm labor assessment algorithm: Low risk of preterm labor. [file kjwhn-2023-08-17-Supplementary-Fig-4.pdf]
